# Immunonutrition—Contributing to the Future of Sustainable Aquaculture by Supporting Animal Performance, Health and Welfare

**DOI:** 10.3390/ani14152275

**Published:** 2024-08-05

**Authors:** Sérgio D. C. Rocha, Cristián A. Valenzuela, Byron Morales-Lange

**Affiliations:** 1 Department of Animal and Aquacultural Sciences, Faculty of Biosciences, Norwegian University of Life Sciences, 1430 Ås, Norway; sergio.rocha@nmbu.no; 2 Grupo de Marcadores Inmunológicos, Laboratorio de Genética e Inmunología Molecular, Instituto de Biología, Pontificia Universidad Católica de Valparaíso, Valparaíso 2374631, Chile; cristian.valenzuela@pucv.cl

Aquaculture is one of the fastest growing food industries worldwide [1]. However, its sustainability has been increasingly scrutinized due to concerns related to the use of wild fish stocks, its environmental impact, and issues associated with animal welfare [1]. In response, researchers around the globe are exploring innovative methods to enhance aquaculture’s efficiency, sustainability and animal robustness. One promising strategy is the development of immunonutritional approaches that can improve fish performance disease resistance and reduce the environmental footprint. As an overview, the current Special Issue (SI), “Sustainable Aquaculture Practices: Novel Feeds to Improve Fish Performance, Immunity and Disease Resistance”, contains 12 articles with a total of 93 authors from 39 institutions spread throughout 14 countries in distinct continents (Figure 1A), focused on how novel functional ingredients and/or bioactive additives can be used during nutritional programming in commercially important aquatic species such as scallop (*Argopecten purpuratus*), Atlantic salmon (*Salmo salar*), coho salmon (*Oncorhynchus kisutch*), rainbow trout (*Oncorhynchus mykiss*), crucian carp (*Carassius auratus*), gilthead seabream (*Sparus aurata*), largemouth bass (*Micropterus salmoides*), meagre (*Argyrosomus regius*), Nile tilapia (*Oreochromis niloticus*), and European seabass (*Dicentrarchus labrax*).

Nutritional programming considers aspects related to the dose of the functional compound, the feeding period, the stage of animal development and season (among others), with the aim of promoting more sustainable aquaculture and animal welfare. Interestingly, a word cloud based on all short summaries and abstracts published in the SI showed that the one of the most used words were fish, diets, growth, proteins, performance, and aquaculture (Figure 1B) related to the following topics: (1) nutrition—ingredient replacement or supplementation, optimization of nutritional requirements in different stages, and improved growth performance; (2) health—immune response, disease management, and animal welfare; and (3) environmental impact and the circular economy—improved efficiency of aquaculture and utilization of byproducts from other industries.

Traditional aquaculture diets often rely on fish meal (FM) and fish oil, which raises concerns due to the depletion of wild fish stocks. In addition, these ingredients are becoming increasingly expensive and subject to price volatility [1]. Thus, there is a growing interest in alternative proteins sources (e.g., plant-based and microbial proteins) and lipid sources for aquafeeds while supporting or improving growth or health. However, different species can tolerate different inclusion levels of novel ingredients, which can deprive the animal of some essential nutrients or introduce anti-nutrients. In both cases, the growth performance can be compromised, and the solution must be species-specific.

Aiming to produce fish in a more sustainable way, Tefal et al. [2] sought to replace FM from sustainable fisheries with organic ingredients as protein sources for juvenile European seabass farming. Here, fish were fed one of several combinations of organic Iberian pig meal byproducts, organic rainbow trout meal byproducts, and insect meal. The results indicated that while fish fed sustainable FM exhibited the highest growth performance, fish fed diets including organic ingredients also showed similar growth and digestibility. However, insect meal was apparently the main contributor to lower fish performance. Therefore, this study suggests that organic protein sources are interesting economical options that can partially replace FM, contributing to improved aquaculture practices.

In an integrative approach between diet and water quality, Welker and Overturf [3] investigated the interaction of fish meal replacements in juvenile rainbow trout reared in a serial reuse water system. The authors used higher doses of different forms of soybean (untreated or processed) as a replacement source of proteins. High levels of fermented soy protein concentrate negatively impacted the growth and survival of rainbow trout when water was used for the third time. The authors also observed some differential expression of stress-related genes (e.g., *fk506*, *dio2*, *regps*, *cyp1a*, *g6ph*, *gadd45a*, and *irf-1* in the liver and gills) associated with diet and water quality. However, when fish were fed higher doses of soy protein concentrate, they demonstrated similar growth to fish fed FM. Hence, this study serves as a relevant baseline for future research where more plant-based diets are used in hatcheries with recirculating water systems.

Still addressing the use of soybean meal in aquaculture, Leeper et al. [4] fed Atlantic salmon with untreated soybean meal (SBM), and two other groups with enhanced SBM, either with enzyme pre-treatment or the supplementation of fructose oligosaccharide. The authors observed that fish fed enhanced SBM displayed improved growth performance. Furthermore, fish fed enzyme pre-treatment SBM showed an increased relative abundance of the lactic acid bacteria *Enterococcus*. Interestingly, for the first time, an interaction between behavior traits and the protein source was described in Atlantic salmon. Fish fed untreated SBM had a possible trend for increased reactive behavior (lower repeatability), which may indicate negative implications for their welfare. On the other hand, fish fed treated SMB displayed a similar behavior to fish fed FM, suggesting the existence of a gut–brain axis related to different protein sources and consequent changes in the intestinal microbiota composition.

This SI also highlights species with a novel commercial interest, such as largemouth bass. Currently, this fish does not have well-defined nutritional requirements at its early life stages. Lukić et al. [5] evaluated different commercial diets and one experiment diet formulation. The selection of diets was based on their content of monounsaturated and polyunsaturated fatty acids, as well as their origin (marine or plant-based). Unbalanced essential fatty acid contents were identified as the possible cause for the observed reduced growth, skeletal development, and/or survival rates. This study is a valuable starting point to design diets with a balanced fatty acid composition aligned with largemouth bass nutritional requirements. Moreover, the role of non-marine ingredients was highlighted, setting the direction for the use of sustainable aquafeeds in this species. Also related to establishing a better feed for the early life stages, the study of Rojas et al. [6] showed that diets rich in highly unsaturated fatty acids from a concentrate of microalgae (*Isochrysis galbana* clone T-iso, *Chaetoceros calcitrans* and *Pavlova lutheri*) can promote growth and disease resistance against bacterial pathogens of hatchery-reared scallop larvae. The authors observed a trade-off between development-associated demands and immune capacity when larvae were exposed to a *Vibrio* outbreak. Specifically, an improvement in cell membrane fluidity and energy metabolic capacity was observed, accompanied by increased expression of immune-related genes (e.g., *aptlr*, *apglys* and *aplbp/bpi1*), which conferred the capacity to control the pathogen virulence. Therefore, by fulfilling the nutritional requirements according to the environment, this study demonstrated that scallop production can be more productive and efficient by using optimal diets.

Optimizing the dietary levels of micronutrients to fulfil the nutritional requirements of fish is a matter of great importance, especially when marine ingredients are replaced by plant-based ingredients. Liu et al. [7] observed that feeding optimal amounts of the mineral manganese (Mn) to post-larval coho salmon led to better growth and manganese accumulation. This mineral is essential to the organism, since is involved in different critical physiological processes like growth, development, and larval survival. In addition, there was a dependency on the manganese present in the diet and the modulation of several signaling pathways, suggesting its involvement in regulating enzymatic activity related to lipid metabolism and antioxidant capacity. Once again, it was shown that nutritional optimization can lead to a more efficient and sustainable production cycle.

Although the effects of novel ingredients can be species-specific, sometimes these compounds may also alter the oxidative state and/or immune homeostasis depending on their inclusion level. These undesired effects can be translated into long-term inflammation, oxidative stress or increased susceptibility to diseases. In addition, during the productive process, aquaculture-related animals are exposed to multi-stressor conditions such as handling, poor water quality, and pathogen exposure, which can further compromise their growth, welfare, and survival. However, several novel ingredients hold the potential to contribute positively to the overall fish health by the modulation of the immune response. It is possible that, when included at an optimal level, novel ingredients can promote immunological robustness, for instance by coordinating mucosa-associated lymphoid tissues or regulating innate immune mechanisms, which can enhance the host’s resistance against pathogens. Consequentially, there is a reduced need for treatments with synthetic antimicrobial compounds (i.e., antibiotics). In this SI, six studies are presented in which feed promoted fish health by using different strategies. Guerreiro et al. [8] assessed the effects of the inclusion of black soldier fly (*Hermetia illucens*) in the diets of meagre, a fish species which high potential for Mediterranean aquaculture diversification. Insects have been identified as an interesting protein source in several fish species that can have a positive effect on the animal’s health. In this study, meagre fed 20% and 30% inclusion levels of black soldier fly did not show a negative compromise in the hepatic and intestinal oxidative status, even though lower growth performance and diet digestibility were detected [8].

However, nutrient replacement is not the only key to promoting aquaculture sustainability; supplementing small amounts of bioactive compounds can also be a determinant factor for healthier animals under production conditions. Chen et al. [9] supplemented crucian carp feed with mulberry leaf (*Morus alba*) extract as a strategy to relieve the effects of stocking density, as well as copper and trichlorfon exposure. The authors identified flavonoids as possible players in the improved growth and reduced oxidative stress of these fish under inappropriate conditions. Although the mode of action requires further exploration, by enhancing digestive enzyme activities and antioxidant status, mulberry leaf extract can support fish health and performance, promoting a more resilient fish. In a similar approach, Teixeira et al. [10] used more targeted compounds to enhance the health of gilthead seabream. Here, the authors used an extract rich in beta-glucans isolated from *Phaeodactylum tricornutum* diatoms and an extract of turmeric (*Curcuma longa*), which is rich in phenolic compounds such as curcumin, to demonstrate their immunomodulatory capacity when fish were fed prior and following an intestinal inflammatory stimulus. The results indicated that beta-glucans and curcumin could mitigate intestinal inflammation and improve overall health at both the intestinal and systemic levels. This supports the beneficial use of natural supplements in fish farming. Furthermore, Yostawonkul et al. [11] supplemented Nile tilapia diets with mangosteen peel (*Garcinia mangostana*) extracts to test this feed additive in different settings of an innovative technological strategy. Their results indicate that fish fed mangosteen peel extracts loaded in a nano-emulsion displayed improved growth performance, immune response, and survival rates during a pathogen challenge with an *Aeromonas veronii* infection. Thus, this approach also contributes to a sustainable aquaculture by using food by-products as a substitute to conventional feed additives and antibiotics.

Another method to promote fish health through feed is by using probiotics, which can have a prophylactic effect against pathogens. Messina et al. [12] supplied gilthead seabream diets with an Archaea species (*Halobacterium salinarum*). It was observed that when challenged with a bacterial pathogen (*Vibrio anguillarum*), fish fed the probiotic had improved growth performance and bactericidal activity. The authors suggested the presence of carotenoids as one of the possible bioactive compounds for the reinforcement of defense mechanisms, such as humoral and cellular immune-related factors, as well as the increase in oxidative burst. The integration of natural immunostimulants not only enhances fish resilience against pathogens but also contributes to reducing the need for antibiotics and other chemical treatments. In a similar approach, Sutthi et al. [13] supplemented Nile tilapia feeds with exopolysaccharides from probiotic *Bacillus tequilensis* PS21 as immunostimulants. The innovative approach used in this study was the use riceberry broken rice, an agro-industrial by-product, as the carbon source for the probiotics. *B. tequilensis* demonstrated antimicrobial properties both during in vitro and in vivo trials, where fish were exposed to *Streptococcus agalactiae*. Also, their data showed an enhanced cellular and humoral response without compromising growth. Therefore, the added value of this biotechnological process promotes a bio-circular economical model and reduces the environmental impact, while at the same time helping to reduce antibiotic dependence and improving fish health.

In summary, the results from the studies published in this SI, “Sustainable Aquaculture Practices: Novel Feeds to Improve Fish Performance, Immunity and Disease Resistance”, outline that immunonutritional strategies using novel feeds are an important alternative approach to contribute to the future of sustainable aquaculture. Depending on the species, novel diets containing functional ingredients and/or bioactive additives can promote animal growth performance, as well as enhance health and welfare (e.g., modulating the host’s immune response, increasing the resistance against pathogens, or contributing to reducing antibiotic application). Nevertheless, continued research and industry efforts and collaboration are pivotal to up-scaling these innovations globally, thus ensuring that aquaculture can meet the growing global demand for seafood while minimizing its ecological footprint.

## Figures and Tables

**Figure 1:**
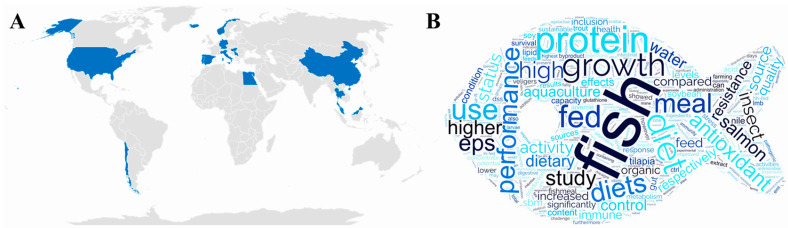
SI overview. (**A**) Distribution of author affiliations (in blue). (**B**) Word cloud created using the combination of all short summaries and abstracts from the SI.

## References

[1] Food and Agriculture Organization (2024). The State of World Fisheries and Aquaculture 2024 Blue Transformation in Action.

[2] Tefal E., Jauralde I., Martínez-Llorens S., Tomás-Vidal A., Milián-Sorribes M.C., Moyano F.J., Peñaranda D.S., Jover-Cerdá M. (2023). Organic Ingredients as Alternative Protein Sources in the Diet of Juvenile Organic Seabass (*Dicentrarchus labrax*). Animals.

[3] Welker T.L., Overturf K. (2023). Effect of Dietary Soy Protein Source on Effluent Water Quality and Growth Performance of Rainbow Trout Reared in a Serial Reuse Water System. Animals.

[4] Leeper A., Sauphar C., Berlizot B., Ladurée G., Koppe W., Knobloch S., Skírnisdóttir S., Björnsdóttir R., Øverland M., Benhaïm D. (2023). Enhancement of Soybean Meal Alters Gut Microbiome and Influences Behavior of Farmed Atlantic Salmon (*Salmo salar*). Animals.

[5] Lukić J., Gyalog G., Horváth Z., Szűcs A.A., Ristović T., Terzić-Vidojević A., Sándor Z.J., Ljubobratović U. (2023). Evaluation of Post-Larval Diets for Indoor Weaned Largemouth Bass (*Micropterus salmoides*). Animals.

[6] Rojas I., Cárcamo C.B., Defranchi Y., Jeno K., Rengel J., Araya M., Tarnok M.E., Aguilar L., Álvarez G., Schmitt P. (2023). A Diet Rich in HUFAs Enhances the Energetic and Immune Response Capacities of Larvae of the Scallop *Argopecten purpuratus*. Animals.

[7] Liu D., Li L., Zhang Q., Yu H. (2023). Effect of Dietary Manganese on the Growth Performance, Lipid Metabolism, and Antioxidant Capacity in the Post-Larval Coho Salmon (*Oncorhynchus kisutch*). Animals.

[8] Guerreiro I., Castro C., Serra C.R., Coutinho F., Couto A., Peres H., Pousão-Ferreira P., Gasco L., Gai F., Oliva-Teles A. (2022). Oxidative Stress Response of Meagre to Dietary Black Soldier Fly Meal. Animals.

[9] Chen G., Long J., Li H., Xu J., Yuan J., Yang Q., Feng L., Wu M., Jiang J. (2023). The Protective Effect of a Dietary Extract of Mulberry (*Morus alba* L.) Leaves against a High Stocking Density, Copper and Trichlorfon in Crucian Carp (*Carassius auratus*). Animals.

[10] Teixeira C., Peixoto D., Hinzmann M., Santos P., Ferreira I., Pereira G.V., Dias J., Costas B. (2022). Dietary Strategies to Modulate the Health Condition and Immune Responses in Gilthead Seabream (*Sparus aurata*) Juveniles Following Intestinal Inflammation. Animals.

[11] Yostawonkul J., Kamble M.T., Sakuna K., Madyod S., Sukkarun P., Medhe S.V., Rodkhum C., Pirarat N., Sewaka M. (2023). Effects of Mangosteen (*Garcinia mangostana*) Peel Extract Loaded in Nanoemulsion on Growth Performance, Immune Response, and Disease Resistance of Nile Tilapia (*Oreochromis niloticus*) against *Aeromonas veronii* Infection. Animals.

[12] Messina C.M., Madia M., Manuguerra S., Espinosa-Ruiz C., Esteban M.A., Santulli A. (2023). Dietary Inclusion of *Halobacterium salinarum* Modulates Growth Performances and Immune Responses in Farmed Gilthead Seabream (*Sparus aurata* L.). Animals.

[13] Sutthi N., Wangkahart E., Panase P., Karirat T., Deeseenthum S., Ma N.L., Luang-In V. (2023). Dietary Administration Effects of Exopolysaccharide Produced by *Bacillus tequilensis* PS21 Using Riceberry Broken Rice, and Soybean Meal on Growth Performance, Immunity, and Resistance to *Streptococcus agalactiae* of Nile tilapia (*Oreochromis niloticus*). Animals.

